# Effects of Stroke on Ipsilesional End-Effector Kinematics in a Multi-Step Activity of Daily Living

**DOI:** 10.3389/fnhum.2017.00042

**Published:** 2017-02-07

**Authors:** Philipp Gulde, Charmayne Mary Lee Hughes, Joachim Hermsdörfer

**Affiliations:** ^1^Institute of Movement Science, Department of Sport and Health Sciences, Technical University of MunichMunich, Germany; ^2^Department of Kinesiology, San Francisco State UniversitySan Francisco, CA, USA

**Keywords:** activities of daily living, apraxia, action disorganization syndrome, kinematics, stroke

## Abstract

**Background**: Stroke frequently impairs activities of daily living (ADL) and deteriorates the function of the contra- as well as the ipsilesional limbs. In order to analyze alterations of higher motor control unaffected by paresis or sensory loss, the kinematics of ipsilesional upper limb movements in patients with stroke has previously been analyzed during prehensile movements and simple tool use actions. By contrast, motion recording of multi-step ADL is rare and patient-control comparisons for movement kinematics are largely lacking. Especially in clinical research, objective quantification of complex externally valid tasks can improve the assessment of neurological impairments.

**Methods**: In this preliminary study we employed three-dimensional motion recording and applied kinematic analysis in a multi-step ADL (tea-making). The trials were examined with respect to errors and sub-action structure, durations, path lengths (PLs), peak velocities, relative activity (RA) and smoothness. In order to check for specific burdens the sub-actions of the task were extracted and compared. To examine the feasibility of the approach, we determined the behavioral and kinematic metrics of the (ipsilesional) unimanual performance of seven chronic stroke patients (64a ± 11a, 3 with right/4 with left brain damage (LBD), 2 with signs of apraxia, variable severity of paresis) and compared the results with data of 14 neurologically healthy age-matched control participants (70a ± 7a).

**Results**: *T*-tests revealed that while the quantity and structure of sub-actions of the task were similar. The analysis of end-effector kinematics was able to detect clear group differences in the associated parameters. Specifically, trial duration (TD) was increased (Cohen’s *d* = 1.77); the RA (Cohen’s *d* = 1.72) and the parameters of peak velocities (Cohen’s *d* = 1.49/1.97) were decreased in the patient group. Analysis of the task’s sub-actions repeated measures analysis of variance (rmANOVA) revealed no impact of the different demands of the sub-actions on the relative performance of the patient group.

**Conclusion**: The analyses revealed kinematic peculiarities in the performance with the ipsilesional hand. These deficits apparently arose from the cognitive demands like sequencing rather than motor constraints. End-effector kinematics proved as a sensitive method to detect and quantify aspects of disturbed multi-step ADL performance after stroke. If standardized, the examination and the analysis are quick and deliver objective data supporting clinical research.

## Introduction

Strokes frequently impair the ability to perform activities of daily living (ADL; Foundas et al., 1995; Forde and Humphreys, 2000; Hartmann et al., 2005; Schwartz, 2006; Wisneski and Johnson, 2007). Stroke related syndromes like apraxia, action disorganization syndrome, hemiparesis and neglect can cause such deficits in ADL. According to a previous estimate 37%–55% of chronic stroke patients are impaired in ADL (Bieńkiewicz et al., 2015).

Following stroke, the behavioral deficits in multi-step ADL arising from impaired action planning are mostly omissions of sub-actions and disorders in the sequencing of subsequent steps, as has been shown in studies on food preparation (Buxbaum, 1998; Schwartz et al., 1999; Bickerton et al., 2007, 2012; Bieńkiewicz et al., 2014), dressing (Sunderland et al., 2006) or hygiene procedures (Humphreys and Forde, 1998). Kinematic analyses do not directly address these types of errors. Kinematics rather quantifies basic aspects of movement execution such as speed, coordination, directness, fluency, smoothness and variability (de los Reyes-Guzmán et al., 2014), although errors can alter kinematic parameters, e.g., the omission of sub-actions can shorten the trajectory of a task or conceptual deficits in handling tools can prolong trial durations (TDs). In this study we consider kinematic measures that can be obtained with only the positional data of the end-effectors.

Up to now, only a few studies employed the approach in the study of multi-step ADL tasks of stroke patients. One investigated scenario is drinking from a glass (Weiss et al., 2000; Alt Murphy et al., 2006, 2011, 2012, 2013; Thies et al., 2009; Kim et al., 2014). Alt Murphy et al. (2011) compared the performance of stroke patients with the performance of age-matched healthy controls. The patients revealed longer movement times, slower peak velocities of the hand and of the elbow (angular peak velocity), a higher number of movement units (less movement smoothness) and an increased trunk displacement when executing the task with their paretic arm. The task was also segmented into five single sub-actions. An analysis of the relative movement times in the different sub-actions showed no differences between healthy subjects and stroke patients indicating that none of the sub-actions was specifically impaired in the patients (Alt Murphy et al., 2011). Notably, parameters representing the patients’ kinematics correlated well with motor function tests like the ARAT, ABILHAND or FMA (Alt Murphy et al., 2012) and reflected changes in motor performance during the first 3 months after a stroke (Alt Murphy et al., 2013). The drinking from a glass task as a multi-step ADL is in comparison to e.g., the tea-making task of moderate complexity (Wood, 1986) since its small set of sub-actions (component complexity) can only be realized in one order (coordinative complexity) and only one of the components changes one of its characteristics (weight of the glass; dynamic complexity).

The present study aimed to examine the feasibility of kinematic parameters when assessing the performance of stroke patients in a complex, multi-step ADL. As a secondary goal we also wanted to gain a better understanding of the reasons underlying impaired ADL performance following stroke. Knowledge of the basic deficits would offer specific targets for interventions in therapy (Bieńkiewicz et al., 2015). Partial results of a pilot of this study have been published in a conference proceeding (Gulde et al., 2014). In order to achieve our main goal we introduced adapted kinematic parameters: relative activity (RA) as a measure of activity, mean peak velocity to describe the average movement speed in tasks with phases of inactivity and number of (velocity) peaks per meter to describe smoothness independent of the amount of executed actions. Such adapted parameters are necessary to analyze irregular signals resulting from the execution of many sub-actions in varying order. The examination of tasks of higher complexity is already being used in the clinical setting, but the assessment is so far qualitative and not quantitative, e.g., trail making tasks or “Multistep Object Use” in the Birmingham Cognitive Screen (BCoS; Bickerton et al., 2012).

The reaction time from the instruction to movement start has been successfully used to quantify the duration of action planning time during simple ADL tasks (Hermsdörfer et al., 2013). During multi-step ADL actions, multiple phases of action planning and movement pauses have to be expected. Consequently, movement pauses were determined with a velocity criterion. The new parameter “RA” resulted, which represents the composition of TD into percentages of activity and inactivity. It is able to indicate prolongations of movement planning and preparation. Such prolongations can be caused by slowed planning of movement trajectories and/or slowed planning of the next action step of a sequence, visual allocation of objects or backwards checking of already performed steps. The second new parameter introduced is the mean peak velocity that quantifies the general movement speed by the average value of action-related velocity peaks.

Considering the literature we expect a number of deviations from normal performance in stroke patients in our ADL-scenario of preparing a cup of tea with milk and sugar, (e.g., Thies et al., 2009; Alt Murphy et al., 2011; Osu et al., 2011; Kim et al., 2014). Due to the, in comparison to e.g., the drinking from a glass task, high complexity (Wood, 1986) of the task (in all three dimensions stated by Wood: component, coordinative and dynamic), we expected the kinematic performance of stroke patients to diverge from the performance of age-matched control subjects. We anticipated increased TDs (Thies et al., 2009; Alt Murphy et al., 2011; Kim et al., 2014) resulting from a reduced movement speed and a higher relative and absolute amount of inactivity. We also expected reduced maximum velocity peaks (Alt Murphy et al., 2011; Osu et al., 2011) and mean peak velocities and reduced movement smoothness (in our study an increased number of (velocity) peaks per meter; Alt Murphy et al., 2011; Osu et al., 2011). Additionally, we hypothesized that path length (PL) may be increased due to sequencing problems and misuse of objects in the patients. Since the sub-actions of the task differ in physical and cognitive demands, we also expected differences in the relative performance of stroke patients in comparison to the age-matched control subjects between the different sub-actions. Such differences can be detected by changes of the relative performance of specific sub-actions (Kim et al., 2014).

## Materials and Methods

### Subjects

Seven patients with lesions following stroke participated in the study (Table 1). In three patients the stroke had affected the right hemisphere right brain damage (RBD) and in four patients the left hemisphere left brain damage (LBD). All patients suffered from hemiparesis. All patients besides patient 6 (Table 1) used their ipsilesional hand for daily activities. Thus RBD patients used their dominant right hand and three of the four LBD patients regularly used their non-dominant left hand. All four LBD patients were aphasic. They were examined for the presence of apraxia with standardized tests of imitation and pantomime (Goldenberg, 1996). The tests included the imitation of hand gestures and finger allocations and production of pantomime with objects of everyday life (e.g., a light bulb). The maximum scores of the tests are dependent on whether a screening version (BCoS) was used or a full version was employed (BCoS; Goldenberg, 1996; Bickerton et al., 2012). Two LBD patients had apraxia according to these tests, the symptoms being moderate in one patient (pathologic in all three tests, but being able to respond correctly to parts of the tasks) and just below the normal range in the other patient (pathologic in one of the three tests). All three RBD patients and one LBD patients showed signs of contralesional neglect. Thus, the patients were quite heterogeneous in their clinical symptoms. Since high variability characterizes typical groups of stroke patients and the association between ADL deficits and clinical symptoms was shown to be only moderate to weak (see “Discussion” Section and e.g., Bieńkiewicz et al., 2015), the patient group was considered adequate for the evaluation of the feasibility of the approach. Exclusion criteria for the patient group were centrally effective medication and/or a bad general condition.

**Table 1:** **Demographic and clinical data for stroke patients and age-matched, elderly healthy control participants (control)**.

Patient	Age in [1a]	Gender	Side of lesion	Month since stroke	Etiology	Neglect	Aphasia	Hemiparesis	Goldenberg/BCoS scores (hand-, finger-imitation)	Goldenberg/BCoS scores (pantomime)	EHI	Degree of hemiparesis
1	70	F	LBD	17.5	Ischemic		Broca	Yes	6/6, 5/6	8/12*	100	2
2	47	F	LBD	12	Ischemic	Right	Global	Yes	2/6*, 3/6*	4/12*	100	2
3	70	M	RBD	72.5	Ischemic	Multimodal		Yes	–		68	2
4	58	F	RBD	36	SAH	Left		Yes	–		100	2
5	79	M	RBD	49	ICH	Left		Yes	–		100	2
6	64	M	LBD	24	Ischemic		Amnestic	Yes (just leg)	20/20, 20/20	53/55	80	1
7	59	M	LBD	4.5	Ischemic		Amnestic	Yes	20/20, 20/20	51/55	88	2
Stroke	63.86 ± 10.38	3F 4M	3RBD/ 4LBD	30.8 ± 23.7							91 ± 13	
Control	70.15 ± 7.12	8F 6M	8 right hand/ 6 left hand								83.2 ± 41.51	

*F, female; M, male; LBD, left sided brain damage; RBD, right sided brain damage; ischemic, ischemic stroke; SAH, subarachnoidal hemorrhage; ICH, intracerebral hemorrhage; Goldenberg/BCos Scores for hand imitation and finger imitation; EHI, laterality quotient according to Edinburgh Handedness Inventory (Oldfield, 1971); the degree of hemiparesis is defined as the following: 1 = no paresis: no impairment, 2 = moderate paresis: impaired, but able to use the hand to hold objects, 3 = severe paresis: not able to functionally move the hand. The assessment of the degree of hemiparesis was based on self-reports and observations. Apraxia was tested either as part of the BCoS Screening Test (Bickerton et al., 2007) or with the tests suggested by Goldenberg (1996); and Goldenberg et al. (2007). The second value denotes the maximum score, asterisks denote a score below the tests’ distribution (pathologic score)*.

Fourteen neurological healthy individuals served as control participants. Exclusion criteria for the control participants were acute or chronic neurologic or psychiatric diseases/disorders, centrally effective medication and/or a bad general condition. In all participants hand preference (in patients before the stroke) was determined with the Edinburgh Handedness Inventory (Oldfield, 1971).

The patients where part of the EU STREP project CogWatch (FP7-ICT-288912) and results of a partially overlapping sample were already published in a conference proceeding (Gulde et al., 2014). Patients were recruited from the former Neuropsychological Unit of the Klinikum München Bogenhausen hospital in Munich, Germany. Ethical approval was obtained by the local ethics committee of the University Hospital Klinikum rechts der Isar in Munich. Informed consent was obtained from all participants. The study was conducted in accordance to the Declaration of Helsinki.

### Set-Up and Procedure

**Figure 1:**
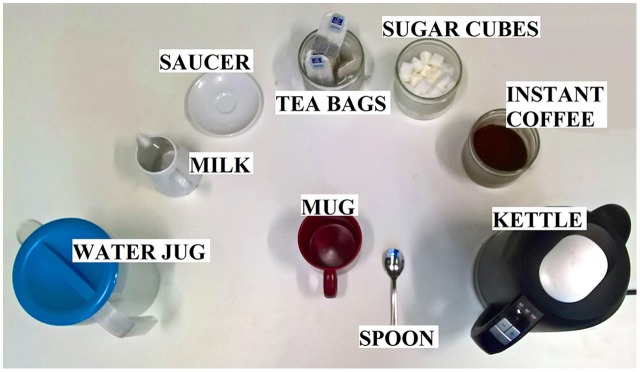
**Experimental set-up: arrangement of the objects on the working surface**.

Participants were asked to unimanually produce a cup of tea with milk and one sugar cube, standing behind a table. They were further asked to perform in a natural way, with no emphasis on speed or perfection. Located on the working surface were the following objects: a water container, a milk carafe, a saucer for used teabags, a jar of teabags, a jar of sugar cubes, an empty kettle, a mug, a spoon and a jar of instant coffee (which was used as a distractor item; Figure 1). Stroke patients performed the task with the ipsilesional hand (the non-paretic hand), while the paretic hand remained inactive, did not participate in the execution of the task, respectively. The use of the dominant and non-dominant hand in the control group was matched to the relation of RBD (3) and LBD (4) patients in the patient sample. This resulted in eight control participants using their dominant and six participants using their non-dominant hand, since one of the LBD patients only suffered from hemiparesis in the leg and this patient preferred to use the dominant hand. The set-up on the table was constant and not adjusted to the used hand. All participants were able to produce the requested cup of tea. Typically, subjects performed two trials. Patients performed a total of 14 and control participants a total of 28 successful trials.

The task was executed unimanually in order to have a performance in that kinematic particularities are not dependent on the degree of paresis but on higher order origin.

Employing an additional analysis of the sub-actions of the tea-making task, we took into account the various degrees of cognitive and motor demands that are connected with the different sub-goals of the task. For example, if a patient has problems in controlling manual interactions with objects, the performance in those sub-actions that demand precise grasps or the manipulation of objects would show peculiarities in the relative kinematics. Such peculiarities could be an increased movement time or PL, for these particular sub-actions. If a patient would suffer from conceptual deficits this also would have effects on the relative kinematics of particular sub-actions that hold for example the handling of complex objects like specialized tools or opening mechanisms.

The task end-goal can be separated into eight sub-actions (Hughes et al., 2013):

1. Pour water in the kettle
2. Switch the kettle on
3. Place a teabag in the mug
4. Pour the heated water into the mug
5. Remove the teabag
6. Add milk
7. Add one sugar cube
8. Stir the tea.

Positional data of the subject’s hand were recorded via a Qualisys motion capturing system using five Oqus cameras (4× Oqus 500 plus and 1× Oqus 510 plus, Qualisys Inc., Gothenberg, Sweden) at a sampling rate of 120 Hz and the Qualisys track manager software (version 2.10 1970). Three passive, reflective markers (diameter 14 mm) were attached to the anterior third of the dorsum of the hand. The marker with the best capturing coverage was later used for the kinematic analysis. In addition, each trial was recorded using digital video at a sampling rate of 30 Hz.

The positional data was processed via MatLab (MATLAB R2011b, MathWorks, Natick, MA, USA). After differentiation of the positional data, the resulting velocity profile was smoothed using a 1 s “Loess” filter (local regression).The partitioning of the task into the described sub-actions was done in two steps. First, coarse boundaries of the sub-actions were defined via video and then fine adjustments were done via the velocity profiles of the hand.

### Non-Kinematic Variables

The video data was used to analyze action errors during task performance. Three significant error types were identified according to Hughes et al. (2013):

- **Misestimation (ME)**

*ME errors are defined as “using grossly too much or too little of some substance”; for example pouring hot water into the mug so that it is only half full*.

- **Execution (EX)**

*EX errors are defined as “an error in the execution of the task”; for example knocking over the mug when reaching for the sugar*.

- **Object substitution (OS)**

*OS errors are defined as “an intended action carried out with an unintended object”; for example using the spoon to add milk*.

### Transition Matrices

As with most ADL, the task end-goal in the tea-making task can be achieved regardless of whether particular sub-actions are omitted, added or performed in a different order (e.g., the cup of tea can be successfully made regardless of whether milk is poured into the cup before adding the sugar cube, or whether the sugar cube is added first).The variability in the task order often observed in ADL tasks provides the opportunity to evaluate the number of performed sub-actions and the resulting sub-action transitions.

Transition matrices (Lames and McGarry, 2007) were produced to describe the probabilities of transitions between different sub-actions. The strength of the 10 × 10 combinations (derived from eight sub-actions plus start and end) is based on the statistical probabilities of behavior of the groups during the task execution.

### Kinematic Variables

**Figure 2:**
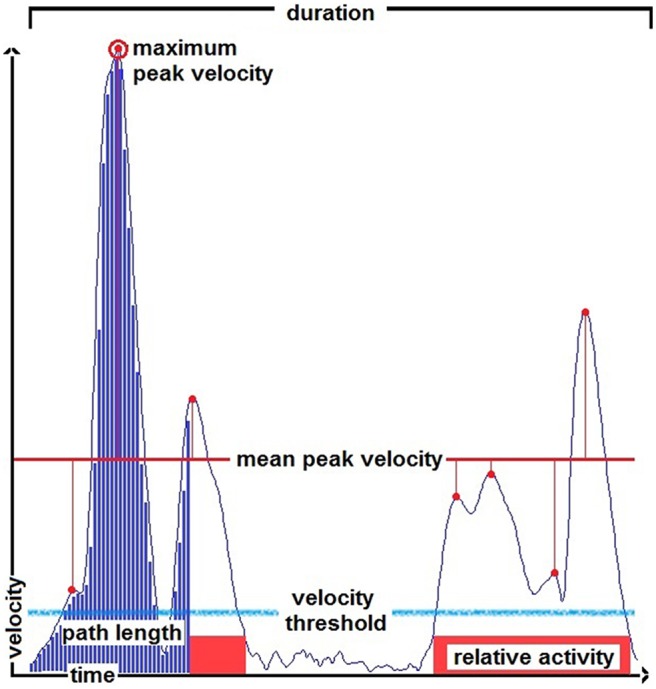
**Illustration of the kinematic variables duration, path length (PL), mean peak velocity, maximum peak velocity and relative activity (RA) indicated in a smoothed velocity profile of a pouring action.** The blue bars scheme an integral, the light blue line indicates a velocity threshold for the determination of velocity peaks, the red boxes indicate the parts of the velocity profile above a velocity threshold used to define the active phases for the calculation of the parameter RA.

The kinematic variables used to describe and compare the patients’ and control participants’ performance during the whole trial as well as during single sub-actions were the following and are, with the exception of the number of (velocity) peaks per meter, displayed in Figure 2:

- **Trial duration (TD) [1 s]**

Time taken to perform the task/sub-actions (time for the heating of the water was excluded).

- **Path length (PL) [1 m]**

Distance traveled by the corresponding end-effector.

- **Maximum peak velocity (VP) [1 m/s]**

Maximal tangential speed reached in the task/sub-actions.

- **Mean peak velocity (MP) [1 m/s]**

Average of the velocity maxima over a certain threshold (0.2 of the mean of the two highest velocity peaks in the whole trial with a minimum of 0.07 m/s) as an indicator of general movement speed independent of breaks made.

- **Number of (velocity) peaks per meter (NP) [1 peak/m]**

The number of velocity peaks over a certain threshold (0.2 of the mean of the two highest velocity peaks in the whole trial with a minimum of 0.07 m/s) as a measure of smoothness related to PL for a temporal and spatial independent measure.

- **Relative activity (RA)**

The ratio of the time the hand was moving and the total TD. The hand was considered to move when velocity exceeded a certain threshold (0.05 m/s). A constant threshold was used to assure the exclusion of noise.

### Statistical Analysis

For the analyses of the kinematic parameters in the trial in total, two-tailed *t*-tests for independent samples were used. The analysis of sub-actions was done via repeated measures analysis of variance (rmANOVA) based on *z*-scores with the control group as the basis, in order to being able to compare the sub-actions to each other. The design was set to “Group” × “Sub-Action” (2 × 8) with the between-subject factor “Group”. *Post hoc*, in the case of significance, *t*-tests were applied and effect-sizes were calculated with Cohen’s *d* (Cohen, 1988). The behavioral data in terms of sub-action performed per trial were analyzed via two tailed *t*-tests for independent samples. To produce the heat-maps of the transition-matrices for the two groups, control and stroke and a matrix according to the task description, matrices were processed via stretching (×10) and smoothing (moving average of 5 × 5) to facilitate a visual inspection. Additional Pearson correlations were computed to compare the outcome parameters (TD and PL, mean peak velocity and RA, mean peak velocity and maximum peak velocity) in the two groups. The threshold of statistical significance was set to *α* = 0.05.

## Results

### Sub-Actions Per Trial

The number of performed sub-actions per trial was not statistically different between the two groups (*p* = 0.14; Table 2). Control subjects performed an average of 8.11 sub-actions per trial that coincided reasonably with the 8-steps assumed for task completion. Stroke patients tended to perform fewer sub-actions per trial with an average of 7.21. Overall, both groups produced the requested tea in all cases but one, where the sugar was added twice by a control subject.

**Table 2:** **Overview and comparison of performed sub-actions per trial**.

Group	Ø Sub-actions	Minimum	Maximum	Significance
Control	8.11 ± 0.90	6	10	*p* = 0.14
Stroke	7.21 ± 1.32	6	10	

*The p-values are derived from two-tailed t-tests for independent samples. When omitting to stir and to remove the teabag, patients as well as control subjects were able to produce the requested tea within six steps, but the outcome is of reduced quality. The maximum count of step was achieved by repeating sub-actions like adding sugar, switching the kettle on and stirring the tea*.

### Error Occurrences

Error frequencies per trial for the three different error types were similar regardless of whether the task was performed by stroke patients or control participants (Table 3). Errors of misestimation occurred most frequently, with individuals typically filling the mug with an inadequately little amount of heated water. Execution errors occurred much less frequently and OS errors were only observed in one trial of control participants.

**Table 3:** **Error frequencies per trial in the groups**.

Group	ME	EX	OS	Sum
Control	0.32 ± 0.46	0.21 ± 0.26	0.07 ± 0.27	0.61 ± 0.53
Stroke	0.43 ± 0.45	0.14 ± 0.24	0 ± 0	0.57 ± 0.45
*p*-value	0.62	0.55	0.34	0.87

*ME, errors of misestimation; EX, errors of execution; OS, errors of object substitution. The p-values are derived from two-tailed t-tests for independent samples*.

### Sub-Action Transition

**Figure 3:**
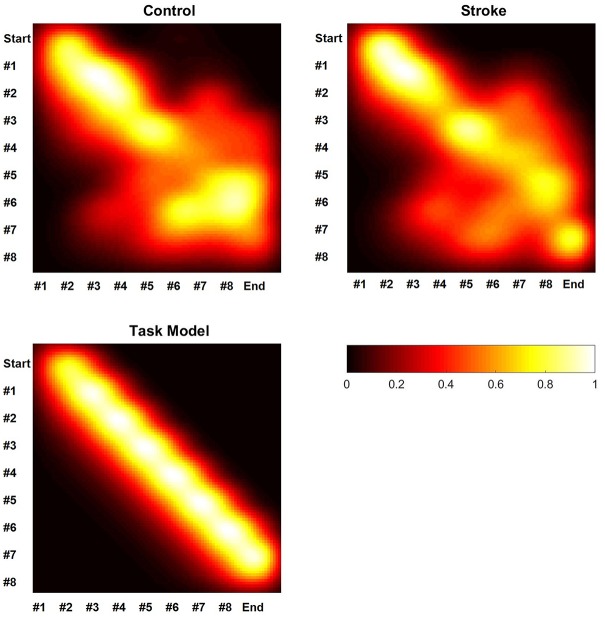
**Schematic heat-maps of the sub-action-transition matrices.** White corresponds with high transition strength and black corresponds with low transition strength. Abscissae and ordinates indicate the number of the sub-action, where the abscissae follow the ordinates during the task execution. The lower part of the figure is showing the schematic heat-map of a hypothetical execution in accordance to the task description.

The graphical illustrations of the transitions between sub-actions in the two groups are shown in Figure 3. The coefficient of the Pearson correlation between the patterns between stroke patients and controls was 0.87 (*p* < 0.01), between the patients’ pattern and the pattern according to the task description was 0.71 (*p* < 0.01) and between the control group and the pattern according to the task description was 0.74 (*p* < 0.01).

### Kinematics: The Task in Total

#### Feasibility

Given the comparable task performance (the number of performed sub-actions and errors per trial) it was possible to analyze potential differences in kinematics.

#### Results

As shown in Table 4 the TD, RA, maximum peak velocity and mean peak velocity differed significantly between the groups.

**Table 4:** **The results from the kinematic analysis of the task in total**.

	Stroke	Control	*p*-value
Trial duration	118.9 s ± 30.4 s	80.2 s ± 17.7 s	0.01, effect size = 1.77
Relative activity	0.63 ± 0.09 activity: 73.6 s ± 11.00 s, inactivity: 45.3 s ± 21.77 s	0.75 ± 0.06 activity: 60.0 s ± 11.7 s, inactivity: 20.2 s ± 7.6 s	0.01, effect size = 1.72 0.02, effect size = 1.19 0.02, effect size = 1.96
Path length	17.8 m ± 1.5 m	17.0 m ± 1.6 m	0.29
Maximum velocity peak	1.00 m/s ± 0.26 m/s	1.31 m/s ± 0.18 m/s	0.02, effect size = 1.49
Mean peak velocity	0.45 m/s ± 0.11 m/s	0.62 m/s ± 0.08 m/s	<0.01, effect size = 1.97
Number of (velocity) peaks per meter	2.43 ± 0.52	2.26 ± 0.33	0.46

*The p-values are derived from two-tailed t-tests for independent samples. Cohen’s d is used for the effect sizes (Cohen, 1988)*.

Additional analyses were conducted to estimate whether the two patient groups differed significantly. The LBD and RBD patients were compared via *t*-test (two tailed for independent samples). Impacts on the kinematic performance may have been caused by the use of the non-dominant hand in LBD patients. However, a larger effect seemed unlikely due to the similar distribution of the hand use in the control group. In addition, different objects were located in the contralesional and ipsilesional table space for the two patient groups, since the setup was constant and not adjusted according to hand use. The exploratory data analysis revealed however no statistical difference between the two sub-groups of the patients (*p* > 0.1 for TD, PL, mean peak velocity, maximum peak velocity, number of (velocity) peaks per meter and RA). Due to the very small sample size, this analysis has however to be considered with great care.

### Kinematics: Sub-Actions

**Figure 4:**
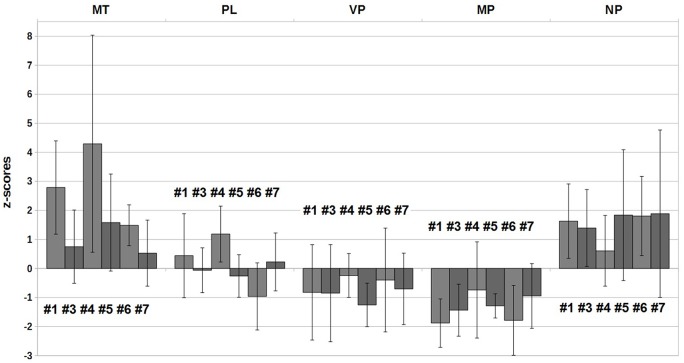
***Z*-scores of the group stroke in the six analyzed sub-actions**.

Using the distribution of the control group as the basis of a *z*-score calculation, we were able to compare the measures between sub-actions and between the different parameters. Due to insufficient data availability, the statistical tests were performed without sub-actions #2 and #8. Sub-action #8 was only rarely used and #2 was in most cases impossible to separate from sub-action #1. As seen in Table 5 and Figure 4, there was at no point a significant impact of the factor “Sub-Action” nor a “Group” × “Sub-Action” interaction.

**Table 5:** **MANOVA of the sub-action performance**.

	Trial duration (TD)	Path length (PL)	Maximum velocity peak (VP)	Mean peak velocity (MP)	Number of (velocity) peaks per meter (NP)
Group	<0.01 *F*_(1,14)_ = 13.958	0.226 *F*_(1,14)_ = 1.605	0.07 *F*_(1,14)_ = 3.784	<0.01 *F*_(1,14)_ = 14.460	<0.01 *F*_(1,14)_ = 23.615
Sub-action	0.76 *F*_(5,70)_ = 0.515	0.84 *F*_(5,70)_ = 0.407	0.22 *F*_(5,70)_ = 1.458	0.86 *F*_(5,70)_ = 0.386	0.42 *F*_(5,70)_ = 1.015
Group × Sub-action	0.87 *F*_(1,14)_ = 0.367	0.69 *F*_(1,14)_ = 0.609	0.11 *F*_(1,14)_ = 1.862	0.53 *F*_(1,14)_ = 0.836	0.22 *F*_(1,14)_ = 1.434

### Correlations

In order to understand the relationship between the different parameters in the two subject groups, correlations were calculated for the parameters in the whole trial. The correlation between TD and PL was calculated to examine if the logical connection “more time, more distance” applies in both groups. The correlation between mean peak velocity and maximum peak velocity was calculated to investigate for a potential redundancy of the commonly used maximum peak velocity and promote the parameter mean peak velocity. Further, the correlation between mean peak velocity and RA was calculated to check for a potential group-related connection between a general movement speed and the ability to perform the task steadily.

**Figure 5:**
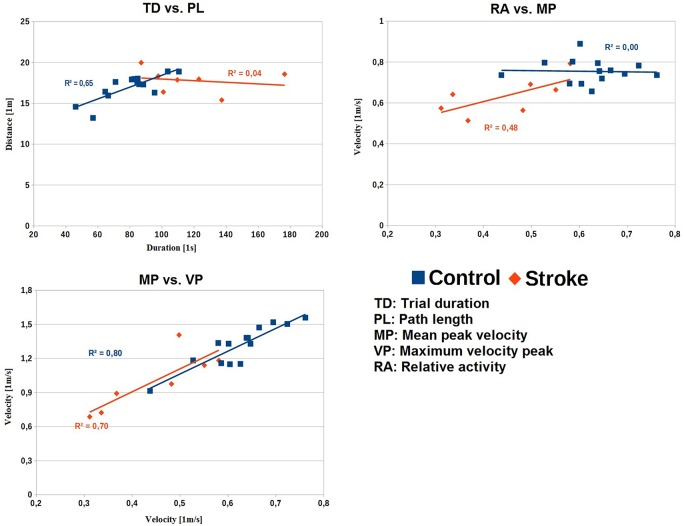
**Correlations with linear trends for the parameter-pairs across the whole task for the two groups.** Upper-left: TD vs. PL. Upper-right: MP vs. RA. Lower-left: MP vs. VP. TD, trial duration [1 s]; PL, path length [1 m]; MP, mean peak velocity [1 m/s]; VP, maximum peak velocity [1 m/s]; RA, relative activity.

As seen in Figure 5, TD was positively correlated with PL for the control group (*r* = 0.80, *p* < 0.01). In contrast, there was no correlation between these two variables for the stroke group (*r* = −0.20, *p* = 0.67). The mean peak velocity revealed a trend to be positively correlated with the RA for the stroke group (*r* = 0.69, *p* = 0.09). In contrast, there was no correlation between these two variables for the control group (*r* = −0.04, *p* = 0.90). The maximum velocity peak for both groups correlated strongly with the mean peak velocity (control *r* = 0.90, *p* < 0.01; stroke *r* = 0.84, *p* = 0.02).

## Discussion

In the present study we examined the end-effector kinematics of chronic stroke survivors during the ADL task of making a cup of tea. The general kinematics differed between patients and age-matched control participants, even though our sample of stroke patients used their non-paretic ipsilesional arm, and did not show clear action errors indicative for action disorganization syndrome. Specifically, patients took longer to complete the tea-making task than control participants, and exhibited a lower mean peak velocity than their age-matched counterparts. Additionally, the longer amount of time that it took for stroke patients to complete the task was predominantly due to an increase in the amount of time in which the hand was inactive.

The statistical analysis of the non-kinematic aspects revealed no statistically significant differences between stroke patients and the group of control subjects in terms of the average number of sub-actions per trial and frequency of errors. The error frequencies in the different sub-actions of the task showed comparable peculiarities for both groups, mainly omitting to remove the teabag and to stir the tea, which was not fatal for the success of the task. The most frequent observed errors were misestimations with the filling level usually being too low when filling the mug. Interestingly the stroke group showed no OS errors, although these errors are frequently observed in stroke patients with similar etiologies (Bieńkiewicz et al., 2015). The transition patterns of the groups both showed quite stable transitions from one sub-action to the next in the beginning of the task and with task progression the performance became more variable.

Therefore, the patients of the sample did not show severe errors of task execution that may have prevented them from completing the task. Such fatal errors have been reported in stroke patients with similar etiologies and symptoms (Forde and Humphreys, 2000; Bieńkiewicz et al., 2015). Two of the patients were apraxic as revealed from tests of imitation and pantomime, and some deficits in the ADL tasks were expected. In addition, detailed analyses of the performance of aiming movements and of motor learning paradigms have revealed a deteriorating impact of apraxia on various aspects of goal direct movements (Haaland et al., 1999; Hermsdörfer et al., 2003; Mutha et al., 2010, 2011). However, the relationship between apraxia as well as other neuropsychological deficits such as aphasia, neglect or inattention and the performance in ADL is not very strong (Hartmann et al., 2005; Schwartz, 2006; Bieńkiewicz et al., 2015). The performance of patients suffering from apraxia or from neglect in the present sample did not deviate strongly from the other patients. All were able to produce the required cup of tea yet revealed at least some kinematic abnormalities. Indeed, the reasons for kinematic ADL deficits seem multifactorial and cannot be concluded from neuropsychological tests or elementary motor performance. Therefore, ADL performance following stroke has to be examined directly. Despite the absence of severe forms of action disorganization syndrome, kinematic analyses revealed clear differences between patients and control subjects. And this was the case despite the ipsilesional, non-paretic hand of the patients was tested. An increase of motor deficits in more demanding situations of execution have been so far reported under dual-task conditions (Regnaux et al., 2005; Houwink et al., 2013). In these studies the authors suggest that with rising demands (in these cases the introduction of a second task) the limited cognitive capacities of stroke patients lead to clear performance decreases in the original motor task. One has to keep in mind that the ADL of tea-making, as complex as it may be, is not demanding maximal motor performance from the subjects and still changes in the kinematic performance were recognized.

Against the original expectation, but in accordance with previous results from a pilot study (Gulde et al., 2014), PLs of the stroke and the control group did not differ. The demands of a complex task did obviously not influence the path. Preserved PL is in accordance with the results for simpler tool use tasks (Clark et al., 1994; Hermsdörfer et al., 2006, 2013). A comparable PL implies that the spatial aspect of movement economy in performing the action-sequence was not affected in the stroke group.

The overall maximum velocity peak was decreased in the stroke patient group compared to the healthy control group, which fits well with the literature (Poizner et al., 1990; Hermsdörfer et al., 1999, 2006; Laimgruber et al., 2005; Tretriluxana et al., 2009). The maximum velocity peak only revealed significant differences in respect to the group but showed not dependence on sub-actions or a “Group” × “Sub-Action” interaction. The maximum peaks of the whole task were more or less randomly scattered over the sub-actions and not associated to a particular one. While the maximum peak velocity is a well-suited parameter for the analysis of single movements, like reaching, its value in more complex analyses is questionable. The mean peak velocity was, as expected, decreased in the patient group. A generally decreased movement speed may be due to slowed actions caused by cognitive demands or by a reduced physical ability to move faster. If velocity is limited by a loss of muscular power, a greater deficit would be expected for the maximum velocity peak than for the average peak velocity and for the velocity parameters a “Group” × “Sub-Action” interaction should be observed. This is however not the case and the relationship between both velocities is the same as in healthy subjects (Figure 5). In addition, subjects were not instructed to move as fast as possible, but to move with their preferred speed. Therefore, the underlying mechanisms that led to a reduced maximum velocity peak appear more to be a general reduction of movement speed than a reduced absolute capacity to generate high movement velocities. In tasks that test maximum motor capacity such as maximum accuracy, speed or fastest response times, deficits independent from primary motor impairments are also typically reported (Godefroy et al., 2010).

The relative number of (velocity) peaks showed, against expectations, no significant difference between stroke patients and age-matched controls in the whole task. The reason could be the sub-maximal demands of the task on the motor performance or a smoothness parameter that is not sensitive enough to detect the differences. In the drinking from a glass tasks the ipsilesional side of patients did reveal a decreased smoothness (Alt Murphy et al., 2011; Osu et al., 2011). This is interesting since Alt Murphy et al. (2011) actually used a very similar parameter to the number of (velocity) peaks per meter. Since the analysis of sub-actions did reveal a decreased smoothness of patients, it seems that the parameter could be overweighting large movements in the analysis of the whole trial and patients are more likely to show decreased movement smoothness in smaller movements (amplitudes). Unfortunately now interaction with the sub-factor sub-action was revealed, so it seems to be rather the lack of sensitivity of the parameter than a peculiarity in the patients’ performance.

The RA differed between the patient and the control group. As expected, patients had a lower relative time of activity during the tea-making task, indicating that the duration of inactivity was particularly prolonged. Stroke patients usually show not only motor but also perceptual deficits and prolonged reaction times (Godefroy et al., 2010; Hermsdörfer et al., 2013). The employed ADL is of submaximal motor demands, so the prolonged phases of inactivity could be based on a decreased perceptual ability or impairment movement planning or action sequencing. If a decreased perceptual ability would have a strong influence on the temporal structure of performance, one would expect an impact on sub-actions with higher demands on visuo-motor integration like grasping a sugar cube, which was not observed. The missing changes in the length of trajectories (PL) would speak against a strong impact of impaired movement planning, which would hypothetically leave the action sequencing as a possible factor. Deficits in sequencing under increased task demands are also consistent with slowing in the trail making tasks, especially in the trail making task B (Godefroy et al., 2010).

The TD is strongly increased in the whole task (effect size of 1.77) and the sub-actions. Increased movement time is a typical finding in most studies of simple prehensile movements (Hermsdörfer et al., 1999; Laimgruber et al., 2005; Tretriluxana et al., 2009) as well as in some cyclic tool use tasks (Clark et al., 1994) performed with the ipsilesional, non-paretic hand in stroke patients. In our task the increased TD could be determined by cognitive demands, like the sequencing of the task, since physical limitations seem unlikely by the following reasons. (1) The PL and number of performed sub-actions of both groups was comparable, so the prolonged TD does not result from differences in the trajectories traveled by the patients’ hand. (2) Additionally, the TD did not correlate with the PL in the patient group in contrast to the control group. This decoupling in patients indicates an influence other than a reduced capacity of mechanical power that discriminates the TDs of controls and patients. (3) The missing “Group” × “Sub-Action” interactions indicate that the different demands of the sub-actions, e.g., conceptual or of fine motor control, did not influence the patients’ performance. (4) Further, patients revealed a lower mean peak velocity and a reduced RA, both parameters showing a strong association with the TD. The analysis of the correlation between those two parameters revealed a trend that patients which were generally moving slower also showed reduced levels of activity, but such correlations with a samples size of 12 and 7, respectively, have to be interpreted with care. So, patients could have had prolonged TDs due to prolonged relative and absolute times of inactivity and reduced speed (and therefore prolonged times of movement). Speaking against the increased cognitive demands are the similar transition patterns and the comparable number of errors between the groups. It might have been that parts of the additional time taken by patients was invested in avoiding errors and producing successful action sequences that lead to the requested product, since errors and mis-sequencing can be fatal. However, this assumption needs further proof.

Another reason for a slowing could arise from the need to execute a normally bimanual task with only one hand. This may have been a specific burden for patients particularly for those using the non-dominant hand. Since we neither evaluated bimanual performance here nor tested learning effects, we cannot definitely answer whether this was the case. However, due to chronic paresis (>6 months in six of the seven patients), we assume that patients had ample experience using only one hand and this may have compensated deficits due to the transition. Rather control subject had to switch without practice and their performance may have deteriorated. This could have produced some of the execution errors reported above and also could have affected some of the kinematic measures. Although we do not see clear indications for such effects, the patients’ deficit may therefore have been in fact underestimated.

In summary, the newly introduced methods of kinematic analysis of ADL performance may offer a number of particular advantages. The use of mean peak velocity instead of maximum peak velocity in ADL appears to not only avoid the risks of erroneous single measures but also represents a measure for ADL tasks, which displays the same performance aspect as the maximum peak velocity in repetitive multi-step movements. The parameter RA adds valuable information to TD, indicating the composition of activity and inactivity. This measure is able to differentiate between more physically pronounced and more cognitively pronounced effects on the TD. The number of velocity peaks per meter turned out to be quite variable and seems not well suited to measure smoothness in such complex multi-step tasks. Unfortunately, measures like spectral arc length or jerk seem also only suitable for this extensive data, when analyses are performed on extracted single sub-actions (Deeming, 1975; Balasubramanian et al., 2012). The segmentation of the task provides the opportunity to differentiate between sub-actions of various degrees of cognitive and motor demands as well as the analysis of sequencing by transition matrices. Introducing the unimanually executed tea making task enables an examination of ADL performance in a multi-step task that comprises complexity due to very different sub-actions (pouring, reaching, placing, stirring) as well as due to a variable sequential order that allows non-fatal errors in sequence planning.

Concluding, the analysis of the tea-making task revealed an impaired performance of the stroke patients in comparison to the control group. The main deficits of the stroke patients were probably given by the cognitive demands (e.g., sequencing) of the task rather than motor constraints or conceptual deficits. The patients’ performance revealed a strongly prolonged TD that could be explained by a decreased mean peak velocity in the execution of single actions within the task and by an additional decreased RA. Prolongation of active and inactive phases may reflect a strategy to gain processing time in view of general resource limitations of the patients in ADL.

The analysis of multi-step ADL revealed a picture of the capabilities of patients in everyday life, recommending further examination of multi-step ADL in terms of the kinematic impact of sequencing deficits. Due to our relatively small sample size further investigations seem warranted. The most appropriate parameter for kinematic analyses is the time (TD), with the mean peak velocity explaining the time in actions and the relative time of activity explaining the time between actions. The search for a suitable smoothness parameter needs further investigation. The kinematic analysis of sub-actions supports a more detailed investigation of motor and cognitive deficits, although no impact could be shown in the present small sample. However, the approach did show clear differences between the groups in unimanual (ipsilesional/dominant side) performance and was able to extend kinematic analysis of tool use and simple functional activities to a more complex ADL task by the introduction of adapted parameters.

Our findings suggest that in clinical routine the assessment of performance by kinematic methods can provide an objective evaluation of the patients’ capabilities and that an increased complexity of employed tasks can reveal additional information. It is already possible to afford motion tracking systems with little budget and further developed algorithms can enable objective and quick analyses for the medical staff in hospitals and health care centers.

Finally, future research with more patients and a differentiation between right and left sided brain damage as well as detailed data on the patients’ basic cognitive deficits (apraxia, neglect, attentional deficit syndrome etc.) will provide results that support the clinical understanding of stroke impact. This preliminary research proves the feasibility of the methodological concept and should pave the way for future studies with emphasis on the interpretation of the patients’ performance with elaborated parameters.

## Author Contributions

PG performed the statistical analysis and was the primary composer of the manuscript. CMLH developed the experimental design. PG and CMLH collected all data from the participants. PG and JH designed the set of parameters for the analyses. PG performed the partitioning of the task into the sub-actions. All authors contributed to the coordination of the study and the final manuscript draft.

## Funding

This study was funded by the EU STREP Project CogWatch Seventh Framework Programme (FP7-ICT-288912).

## Conflict of Interest Statement

The authors declare that the research was conducted in the absence of any commercial or financial relationships that could be construed as a potential conflict of interest.
